# Developmental Pharmacogenetics of CYP2D6 in Chinese Children: Loratadine as a Substrate Drug

**DOI:** 10.3389/fphar.2021.657287

**Published:** 2021-07-06

**Authors:** Qian Li, Yue-E Wu, Kai Wang, Hai-Yan Shi, Yue Zhou, Yi Zheng, Guo-Xiang Hao, Yi-Lei Yang, Le-Qun Su, Wen-Qi Wang, Xin-Mei Yang, Wei Zhao

**Affiliations:** ^1^ Department of Clinical Pharmacy, The First Affiliated Hospital of Shandong First Medical University and Shandong Provincial Qianfoshan Hospital, Shandong Engineering and Technology Research Center for Pediatric Drug Development, Shandong Medicine and Health Key Laboratory of Clinical Pharmacy, Jinan, China; ^2^ Department of Clinical Pharmacy, Key Laboratory of Chemical Biology (Ministry of Education), School of Pharmaceutical Sciences, Cheeloo College of Medicine, Shandong University, Jinan, China; ^3^ Department of Pediatrics, The First Affiliated Hospital of Shandong First Medical University and Shandong Provincial Qianfoshan Hospital, Shandong Engineering and Technology Research Center for Pediatric Drug Development, Jinan, China

**Keywords:** children, plasma, ontogeny, pharmacogenetics, CYP2D6

## Abstract

**Objective:** The elucidation of CYP2D6 developmental pharmacogenetics in children has improved, however, these findings have been largely limited to studies of Caucasian children. Given the clear differences in CYP2D6 pharmacogenetic profiles in people of different ancestries, there remains an unmet need to better understand the developmental pharmacogenetics in populations of different ancestries. We sought to use loratadine as a substrate drug to evaluate the effects of ontogeny and pharmacogenetics on the developmental pattern of CYP2D6 in Chinese pediatric patients.

**Methods:** Chinese children receiving loratadine treatment were enrolled in the present study. The metabolite-to-parent ratio (M/P ratio), defined as the molar ratio of desloratadine to loratadine of trough concentrations samples at steady-state condition, was used as a surrogate of CYP2D6 activity. Loratadine and desloratadine were determined by LC/MS/MS method. Variants of CYP2D6 were genotyped by polymerase chain reaction for CYP2D6 *4, *10, *41 and long polymerase chain reaction for CYP2D6 *5.

**Results:** A total of 40 patients were available for final analysis. The mean age was 4.50 (range 0.50–9.00) years and the mean weight was 19.64 (range 7.00–42.00) kg. The M/P ratio was significantly lower in intermediate metabolizers (IMs) compared to normal metabolizers (NMs) (10.18 ± 7.97 vs. 18.80 ± 15.83, *p* = 0.03). Weight was also found to be significantly associated with M/P ratio (*p* = 0.03).

**Conclusion:** The developmental pharmacogenetics of CYP2D6 in Chinese children was evaluated using loratadine as a substrate drug. This study emphasizes the importance of evaluating the developmental pharmacogenetics in populations of different ancestries.

## Introduction

Many age-related physiological and biological factors, such as gastric pH, intestinal motility, liver, intestinal enzymes and transporters, can lead to changes in drug disposition during childhood development (Kearns et al., 2003; Hines, 2008). The developmental changes in cytochrome P450 (CYP) enzyme activity during the growth and development of children has important influences on the differences in age-related drug disposition (de Wildt et al., 2014).

CYP2D6 accounts for about 2% of the total liver CYP content in adults (Shimada et al., 1994), and mediates the metabolism of many drugs, such as analgesics, cardiovascular, antidepressant and antipsychotics (Allegaert et al., 2011). CYP2D6 ontogeny has been published in fetal, pediatric and adult *in vitro* or *in vivo* (Ladona et al., 1991; Treluyer et al., 1991; Jacqz-Aigrain et al., 1993; Tateishi et al., 1997; Hu et al., 1998; Kennedy et al., 2004; Allegaert et al., 2005; Blake et al., 2007; Johnson et al., 2008; Stevens et al., 2008). However, the interpretation of these data is controversial, previous studies showed that CYP2D6 protein and activity increased in the first month of life, reaching approximately two-thirds of adult levels between 1 month and 5 years of age (Treluyer et al., 1991). Other studies have reported that the levels of CYP2D6 protein were similar between infants who were aged 0–1 compared to individual over 1 year of age (Ladona et al., 1991; Tateishi et al., 1997). Notably, CYP2D6 activity has been detected in children 2 weeks old, consistent with genotype, and remained unchanged at 1 year of age (Blake et al., 2007). Some studies observed that CYP2D6 expression increased sharply in the first year of life considering the maturity level of renal function in the first year (Johnson et al., 2008) and CYP2D6 ontogeny is complete by age of 1 year (Hu et al., 1998; Kennedy et al., 2004; Allegaert et al., 2005). For example, several studies have found that in pediatric populations (age 1–8 years) the CYP2D6 normal metabolizers had already reached adult levels of CYP2D6 enzyme activity (Hu et al., 1998; Kennedy et al., 2004; Allegaert et al., 2005). Further studies are required to explore the age-related developmental pattern of CYP2D6 by ancestry.

In addition to age-related factors, pharmacogenetic polymorphisms further contribute to inter-individual differences in drug response. Pharmacogenetic variants in the CYP2D6 gene can result in increased, decreased, or complete loss of CYP2D6 activity (Allegaert et al., 2005; Allegaert et al., 2008; Stevens et al., 2008). The incidence of specific polymorphisms in CYP2D6 can vary widely in different worldwide ancestries. For example, it has been widely reported that the incidence of CYP2D6*10, which reduces the activity of CYP2D6, is carried by approximately 50% of people with Asian ancestry, but is rare in European populations (Wang et al., 1993; Johansson et al., 1994).

Previous studies on developmental pharmacogenetics of CYP2D6 have been well documented in Caucasian population (Kennedy et al., 2004; Allegaert et al., 2005; Blake et al., 2007), alleles such as CYP2D6*3, CYP2D6*4, and CYP2D6*5 accounts for about 98% of the poor metabolizers (Gaedigk et al., 1999). The elucidation of CYP2D6 developmental pharmacogenetics in these populations has improved. While in Asian population, CYP2D6*3 and *4 are relatively rare, and the high frequency of the CYP2D6*10 allele (Wang et al., 1993; Johansson et al., 1994) may lead to the low CYP2D6 activity in Asian normal metabolizers. Thus, there are still some gaps of knowledge in people with Asian ancestry. Given the clear differences in CYP2D6 pharmacogenetic profiles in people of different ancestries, there remains an unmet need to better understand the developmental pharmacogenetics in populations of different ancestries. We hypothesized that both developmental factors and pharmacogenetics (classify by the CYP2D6 phenotypes) can explain the developmental pattern of CYP2D6 in Chinese children. Therefore, we carried out a developmental pharmacogenetic study of CYP2D6 using loratadine as a substrate drug in Chinese children.

The reasons for choosing loratadine as a substrate drug are as follows:

 1) It is often used to treat allergic diseases in Chinese children, such as seasonal allergies and rashes (Clissold et al., 1989).
 2) Loratadine is known to be a substrate for CYP2D6 based on previous *in vitro* studies (Yumibe et al., 1996; Ghosal et al., 2009; Sheludko et al., 2018). CYP2D6 is the enzyme that plays an important role in the pharmacokinetic variability of loratadine in Chinese population. After oral administration, loratadine is absorbed rapidly and undergoes extensive metabolism in the body. One of the main metabolites, desloratadine, is reported to have more pharmacological potencies than loratadine (Geha and Meltzer, 2001; Ramanathan et al., 2007).
 3) The significant effects of CYP2D6 polymorphism on the pharmacokinetics of loratadine has been reported in Chinese adults. For example, loratadine exposure was 123.6% higher (*p* < 0.05) and CL/F decreased by 50.9% (*p* < 0.01) in CYP2D6 wild type Chinese adults compared to homozygous CYP2D6*10/*10 subjects. Similarly, Loratadine exposure was 75.5% higher (*p* < 0.05) and CL/F decreased by 35.2% (*p* < 0.01) in CYP2D6*10 heterozygotes compared to CYP2D6*10/*10 homozygous subjects (Yin et al., 2005).

## Methods

### Subjects

The study was designed and carried out at Shandong Provincial Qianfoshan Hospital. Patients aged from 6 months to 12 years with allergic disorders such as allergic rhinitis or allergic asthma and underwent routine treatment of loratadine were included in the present study. The study protocol was approved by the ethical committees of the clinical institution (Shandong Provincial Qianfoshan Hospital). Informed-consent forms were obtained from the patients’ parents or guardians before the initiation of the study.

Patients receiving loratadine (10 mg; schering plough, Shanghai, China) as part of routine anti-allergy therapy were eligible to be included in the present study. Patients were excluded if they had co-administrated with known inhibitor of CYP2D6 such as fluoxetine, paroxetine and amiodarone. Loratadine was administered as follows: 10 mg once daily for children >30 kg and 5 mg once daily for children ≤30 kg. The elimination half-life of loratadine and desloratadine are 8–14 h and 17–24 h, respectively (Vlase et al., 2007). For plasma concentration determination, patients took loratadine tablets for 10 days to a steady-state condition and blood samples were collected anywhere between 12 and 24 h after the last dose. Only one blood sample was drawn from one participant. The molar ratio of desloratadine to loratadine of trough concentrations samples (the M/P ratio) was used as a surrogate of CYP2D6 activity. Blood samples were centrifuged at 4,000 rpm for 10 min and stored at −80°C until analysis.

### Determination of Loratadine and Its Metabolites

Trough plasma concentrations of loratadine and its main active metabolite desloratadine were determined by liquid chromatography-mass spectrometry (LC/MS/MS) (Li et al., 2020). After mixed with the internal standard (IS, propranolol) and precipitated with methanol, plasma samples were then separated on a C18 column with a gradient mobile phase consisting of water (containing 0.1% formic acid) and acetonitrile. After centrifuged, 20 µl of the supernatants were injected into the HPLC system. The quantitation of loratadine, desloratadine and IS was performed using MRM mode and the transitions were: 383.1 → 337.1 for loratadine, 311.1 → 259.0 for desloratadine and 260.2 → 116.0 for IS, respectively. The calibration curve ranged from 0.20 to 50 ng/ml for both loratadine and desloratadine. The inter-day and intra-day coefficients of variation (CVs) of controls were both below 9.20%. The accuracy of loratadine and desloratadine were 102.50–109.21% and 101.81–108.83%, respectively. The lower limit of quantification (LLOQ, associated inter-day and intra-day CVs) were 0.20 ng/ml for both loratadine and desloratadine.

### Genotyping

DNA was extracted with a TIANamp Blood DNA Kit (TianGen Biotech, Beijing, China) from whole blood sample. The single nucleotide polymorphisms (SNPs) of CYP2D6*4 (rs3892097), CYP2D6*10 (rs1065852), and CYP2D6*41 (rs28371725) were selected for genotyping, primers and probes were obtained from Thermo Fisher Scientific. The polymerase chain reaction (PCR) was performed to identify CYP2D6 alleles on a Fluorescence Quantitative PCR (Bio-Rad, Hercules, CA, United States) according to the TaqMan allelic discrimination assay (Applied Biosystems, Foster City, CA, United States). Long polymerase chain reaction (PCR)-based methods from Hersberger et al. (2000) were employed to detect the CYP2D6∗5 (whole gene deletion).

The consensus CYP2D6 activity score (AS) was used to classify phenotypes according to CPIC and DPWG guidelines (Blake et al., 2007; Gaedigk et al., 2017; Caudle et al., 2020). The patients were divided into two groups NMs (normal metabolizers, 1.25 ≤ AS ≤ 2.25) and IMs (intermediate metabolizers, 0 < AS < 1.25). Patients who did not carry CYP2D6*4, *5, *10, or *41 were assumed to have a CYP2D6 activity score of 2 (CYP2D6*1/*1).

### Statistical Analysis

The statistical analyses were performed using SPSS statistical software version 16.0 (IBM SPSS Statistics, IBM Inc., NY, United States). The medians and ranges of continuous variables were summarized and linear regression analysis was carried out. Mann-Whitney *U* test was used to study the effect of the CYP2D6 phenotypes on the M/P ratio (the molar ratio of desloratadine to loratadine). Multiple regression analysis was performed to determine the association of multiple factors such as weight, age and CYP2D6 phenotypes with M/P ratio. Factors that showed to be significant factors of M/P ratio from bivariate analysis (*p* < 0.05) were selected as candidate covariates in the multivariate test. A *p* value of < 0.05 was considered statistically significant for all tests.

## Results

Forty-three patients were enrolled in this study. Three of them had loratadine and desloratadine levels below the LLOQ (0.20 ng/ml), these cases were excluded from the study since the data cannot be used to calculate the M/P ratio quantitatively. In final, 40 patients were available for final analysis. The mean (standard deviation) age was 4.50 (2.57, range 0.50–9.00) years and the mean (standard deviation) weight was 19.64 (8.13, range 7.00–42.00) kg. The characteristics of patients and data on CYP2D6 phenotypes are summarized in Table 1. The two groups NMs and IMs are comparable in terms of age, weight, gender, dose, and creatinine clearance rate in Table 2.

**TABLE 1:** Characteristics of the 40 children.

Patient characteristics	Number	Mean (SD)	Median (range)
Patients	40		
Gender (Male/Female)	20/20		
Age (years)		4.50 (2.57)	4.00 (0.50–9.00)
Body weight (kg)		19.64 (8.13)	17.50 (7.00–42.00)
Loratadine dose (mg)		5.25 (1.10)	5.0 (5.00–10.00)
Loratadine concentrations (ng/ml)		0.86 (1.44)	0.44 (0.20–8.86)
Desloratadine concentrations (ng/ml)		4.97 (3.38)	4.23 (0.39–15.40)
M/P ratio		13.20 (11.90)	9.93 (0.83–61.33)

Phenotype	Diplotype	AS	Number	Frequency
NMs	*1/*1	2.0	9	22.5%
	*1/*41	1.5	1	2.5%
	*1/*10	1.25	4	10.0%
IMs	*10/*41	0.75	1	2.5%
	*10/*10	0.5	25	62.5%

Range: Minimum value–Maximum Value.

M/P ratio: the metabolite-to-parent ratio, the molar ratio of desloratadine to loratadine.

AS, activity score.

**TABLE 2:** Comparison between NMs and IMs.

Type	NMs	IMs	*p*
Age (years) (mean ± SD)	4.19 ± 2.76	4.67 ± 2.50	0.60
Body weight (kg) (mean ± SD)	17.39 ± 6.27	20.85 ± 8.85	0.32
Cr (µmol/L) (mean ± SD)	29.70 ± 10.98	33.63 ± 11.60	0.15
Gender (Male/Female)	8/6	12/14	0.51
Loratadine dose (5 mg/10 mg)	14/0	24/2	0.24

**FIGURE 1:**
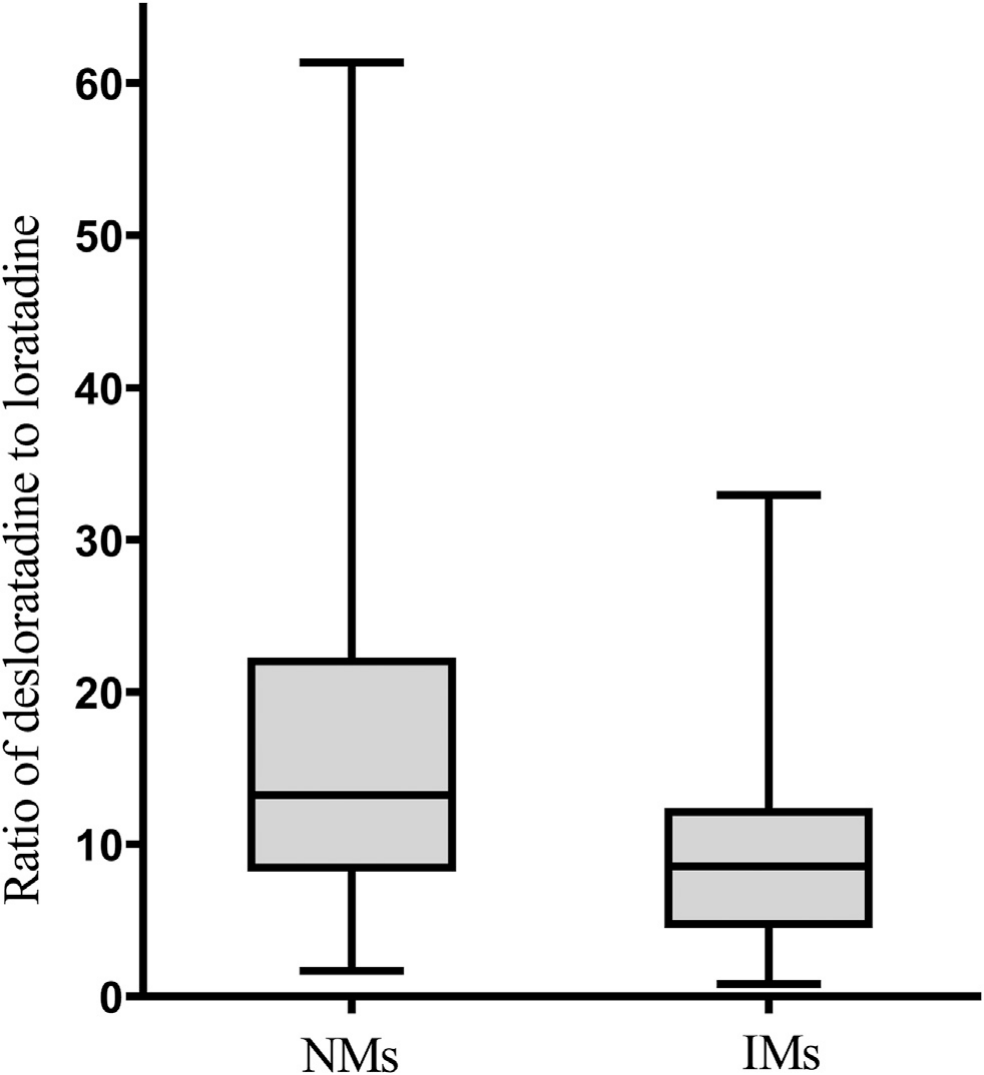
Impact of CYP2D6 phenotypes on the M/P ratio of loratadine. The bars are medians, boxes are interquartile range, whiskers represent the min to max. Significant (*p* = 0.03) difference between NMs and IMs.

**FIGURE 2:**
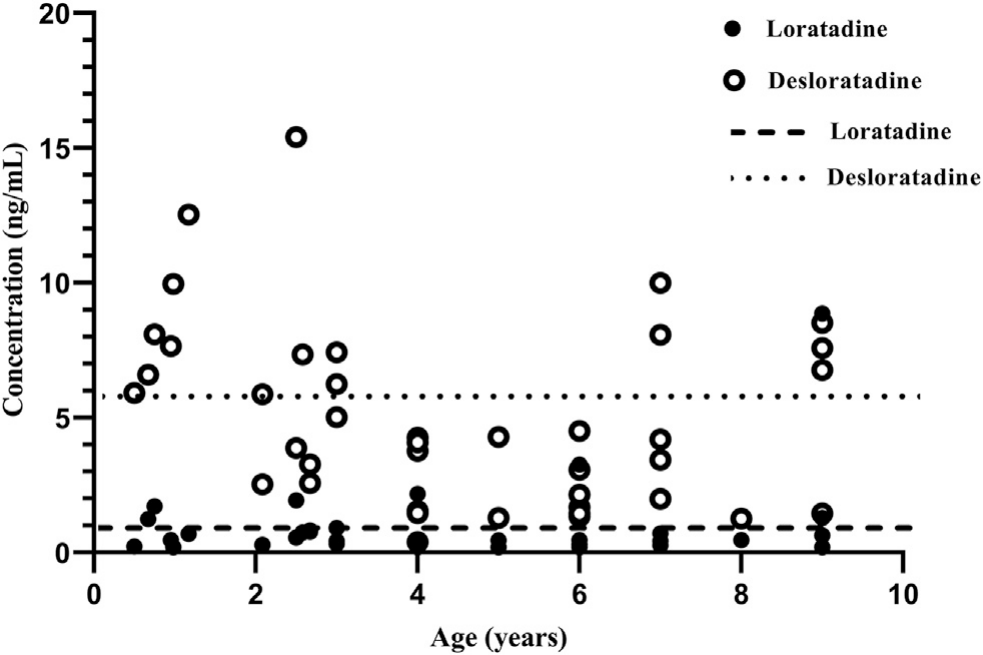
The relationship between plasma concentration vs. age. The long dotted line is the mean trough plasma concentration of loratadine in steady-state condition in adults. The short dash line is the mean trough plasma concentration of desloratadine in steady-state condition in adults.

The M/P ratio was significantly associated with CYP2D6 phenotypes. The M/P ratio was significantly lower in intermediate metabolizers (IMs) compared to normal metabolizers (NMs) (10.18 ± 7.97 vs. 18.80 ± 15.83, Mann–Whitney *U*-test, *p* = 0.03) (Figure 1). Weight was also found to be significantly associated with M/P ratio (*p* = 0.03). There was not a significant association between age and M/P ratio (Figure 2). In final, multiple linear regression analyses revealed distinctive factors included weight and CYP2D6 phenotypes associated with the M/P ratio. These two factors could explain about 19.7% of the interindividual variability in the M/P ratio (*R* ^2^ = 0.197).

## Discussion

The developmental pharmacogenetics of CYP2D6 was evaluated for the first time in Chinese children. The impact of CYP2D6 phenotypes on CYP2D6 activity was demonstrated using a substrate drug of loratadine.

Previous studies on pharmacogenetics of CYP2D6 have been well documented in Caucasian population. Several alleles of the CYP2D6 such as CYP2D6*3, CYP2D6*4, CYP2D6*5, and CYP2D6*10 that cause absent or decreased enzyme activity have been reported (Gaedigk et al., 1999). In Caucasian population, CYP2D6*3, CYP2D6*4, and CYP2D6*5 alleles accountes for about 98% of the poor metabolizers (Gaedigk et al., 1999), while in Asian population, CYP2D6*3 and *4 are rarely found, and the frequency of the CYP2D6*5 alleles is about 5% (Wang et al., 1993). Although the frequency of the CYP2D6*2 alleles is high anywhere between 10 and 50% (Gaedigk et al., 2017), CYP2D6*2 is currently considered to be a normal function allele by CPIC and DPWG; however, this function assignment has been challenged and some laboratories report CYP2D6*2 function differently. Function of this allele will be reassessed as additional data become available (Kearns et al., 2003; Hines, 2008). Meanwhile, the CYP2D6*10 allele occurs in approximately 50% of Asian population, but is extremely rare in white population (Wang et al., 1993; Johansson et al., 1994). Therefore it has been postulated that the high frequency of the CYP2D6*10 allele causes the low CYP2D6 activity in Asian normal metabolizers. In this study, the M/P ratio was significantly lower in IMs than those in NMs. These results suggest the CYP2D6 activity is poor in IMs subjects (60.46% subjects were CYP2D6*10/*10 genotypes), which is similar to that reported in adults (Yin et al., 2005). Additionally, similar observations were made in the relationship between the CYP2D6*10 genotype and the pharmacokinetics of haloperidol in Asian subjects (Mihara et al., 1999; Roh et al., 2001). Therefore, our present study suggest that the CYP2D6 polymorphisms with CYP2D6*10/*10 plays an important role in lower the activity of CYP2D6 in Chinese adults and children.

Our results showed no significant association between M/P ratio and age. This may be related to the relatively small sample size. Based on conclusion of most previous research (Kennedy et al., 2004; Allegaert et al., 2005; Blake et al., 2007; Johnson et al., 2008; Stevens et al., 2008; de Wildt et al., 2014), the levels of CYP2D6 enzyme activity were similar between children over 1 year of age and adults. For example, *in vitro*, CYP2D6 enzyme activity is low before birth, but increases to adult levels in the first weeks or months after birth (de Wildt et al., 2014). *In vivo*, CYP2D6 activity was detectable and consistent with genotype by 2 weeks old, no change was found in the first year after birth (Blake et al., 2007). In pediatric population (range 1.1–8.0 years), CYP2D6 activity was similar with adult level (Kennedy et al., 2004; Allegaert et al., 2005). We compared the trough plasma concentrations of loratadine and desloratadine to adult levels in steady-state condiition (Figure 2). The result is similar to adults (Radwanski et al., 1987). Due to the limited number of patients, there were only four individual aged 6 months to 1 year, and future research should include a larger patient sample population to accurately identify the correlation between age and CYP2D6 pharmacogenetics in Chinese pediatric population.

Our study did not use the “golden” substrate drugs of CYP2D6 (i.e., dextromethorphan and metoprolol) (Frank et al., 2007), as these drugs were rarely used in pediatric routine practice in China. Loratadine was selected in our present study, not only because of its routine use and determinant role of CYP2D6 on its metabolism, but also the ethical reason. Although CYP3A4, CYP2D6, and CYP2C19 are involved in the metabolism of loratadine (Yumibe et al., 1996; Ghosal et al., 2009), the efficiency of loratadine conversion by CYP2D6 was 4.5∼5-fold higher than that of CYP3A4 (Yumibe et al., 1996; Ghosal et al., 2009; Sheludko et al., 2018) and CYP2D6*10 allele accouts for approximately 50% in Asian subjects (Johansson et al., 1994). For CYP3A4, many variant alleles such as CYP3A4*1B, *2, and *3 are absent in Chinese subjects (Walker et al., 1998; Sata et al., 2000; van Schaik et al., 2001). Some Asian-specific alleles such as CYP3A4*4, *5, *18, and *19, have been reported with frequencies of 1–3% (Dai et al., 2001; Hsieh et al., 2001), but the functions of these alleles *in vivo* are still uncertain (Eiselt et al., 2001). For CYP2C19, the contribution of the formation of the major circulating metabolite desloratadine was minor (Ghosal et al., 2009). Based on current information, the clinical importance of CYP3A4 and CYP2C19 polymorphisms are not likely for the majority of Chinese population. As the loratadine followed a one-compartment elimination, the trough concentrations could be a reliable surrogate of drug exposure and elimination (Yin et al., 2005). Given the unmet need of understanding developmental pharmacogenetics of CYP2D6 in inter-ancestry population, an adaptive substrate drug should be considered in pediatric pharmacology study (Zhao et al., 2018).

Our study collected trough plasma samples to determine the metabolic ratio of loratadine, because the distribution of drug in plasma, tissues and urine was in equilibrium at multiple-dose steady-state. Studies have shown that the effect of age and CYP2D6 activity scores on plasma M/P ratio interindividual variability is similar to that of 24 h urinary M/P ratio (Allegaert et al., 2008). Besides, studies documented that the sensitivity of CYP2D6 activity *in vivo* was different based on urine metabolic ratio’s to urine pH (Stamer et al., 2003). As the volume and the number of samples that can be taken at once are limited in children, we did not choose the AUC approach in the study. Based on these reasons, we selected the molar ratio of desloratadine to loratadine of trough concentrations samples as a surrogate of CYP2D6 activity.

Our research had some limitations. Firstly, since the primary objective of this study was to describe the developmental pattern of CYP2D6, clinical outcomes were not evaluated. Developmental pharmacokinetics and pharmacodynamics need to be evaluated in the following studies. Secondly, CYP2D6 gene duplication such as CYP2D6*1xN, *10xN, and *36xN had the lower frequency in Chinese people and these structural variants were not analyzed (Gaedigk et al., 2017). This is a limitation since 10xN and *36xN with decreased function may have some impact in those patients that carry it (Del Tredici et al., 2018). Meanwhile, due to the limited number of patients, we did not found CYP2D6*4, CYP2D6*5 in this population, and future research should include a larger patient sample population to accurately identify the correlation between CYP2D6*5, CYP2D6*41, CYP2D6*10, and gene duplication polymorphisms and CYP2D6 pharmacogenetics in Chinese pediatric population. Thirdly, the unexplained variability in our study is still large, indicating that other covariates contribute to the pharmacokinetics of loratadine. These missing covariates also require further evaluation. In conclusion, the developmental pharmacogenetics of CYP2D6 in Chinese pediatric patients was evaluated using loratadine as a substrate drug. CYP2D6 phenotypes showed independently significant impact on M/P ratio. Our results emphasize the importance of evaluating the developmental pharmacogenetics in populations of different ancestries.

## Data Availability

The raw data supporting the conclusions of this article will be made available by the authors, without undue reservation, to any qualified researcher.
